# Patient-Reported Outcomes and Factors Impacting Success of the Periacetabular Osteotomy

**DOI:** 10.7759/cureus.37320

**Published:** 2023-04-09

**Authors:** Muzammil Akhtar, Daniel I Razick, Jimmy Wen, Rafaay Kamran, Ubaid Ansari, Khizur Kamran, Ramy Khalil, Burhaan Syed, Muhammad Karabala, Sarah Preiss-Farzanegan

**Affiliations:** 1 Surgery, California Northstate University College of Medicine, Elk Grove, USA; 2 Physical Medicine and Rehabilitation, California Northstate University College of Medicine, Elk Grove, USA; 3 Molecular Environmental Biology, University of California Berkeley, Berkeley, USA; 4 Internal Medicine, California Northstate University College of Medicine, Elk Grove, USA

**Keywords:** hip dysplasia, western ontario and mcmaster universities arthritis index (womac), modified harris hip score, harris hip score, periacetabular osteotomy

## Abstract

Hip dysplasia is a condition affecting both infants and adults, characterized by a shallow acetabulum that does not sufficiently cover the head of the femur. This leads to instability of the hip and elevated levels of mechanical stress around the acetabular rim. A popular procedure for the correction of hip dysplasia is the periacetabular osteotomy (PAO), in which fluoroscopically guided osteotomies around the pelvis are made to allow for repositioning of the acetabulum to fit properly on the femoral head. This systematic review aims to analyze patient factors that impact outcomes, as well as patient-reported outcomes such as the Harris Hip Score (HHS) and the Western Ontario and McMaster Universities Arthritis Index (WOMAC). The patients in this review did not undergo any prior intervention for acetabular hip dysplasia, allowing for an unbiased reporting of outcomes from all included studies. Of studies reporting HHS, the mean preoperative HHS was 68.92 and the mean postoperative HHS was 89.1. Of the study that reported mHHS, the mean preoperative mHHS was 70, and the mean postoperative mHHS was 91. Of the studies reporting WOMAC, the mean preoperative WOMAC was 66, and the mean postoperative WOMAC was 63. Key findings of this review are that of the seven included studies, six achieved a minimally important clinical difference (MCID) based on patient-reported outcomes, and factors impacting outcome are preoperative Tönnis osteoarthritis (OA) grade, pre and postoperative lateral-center edge angle (LCEA), preoperative hip joint congruency, postoperative Tönnis angle, and age. In patients with no prior intervention for hip dysplasia, the PAO is a successful procedure with significant improvement in postoperative patient-reported outcomes. Despite the reported success of the PAO, proper patient selection is vital to avoid early conversions to total hip arthroplasty (THA) and prolonged pain. However, further investigation is prompted regarding the long-term survivorship of the PAO in patients with no prior intervention for hip dysplasia.

## Introduction and background

Hip dysplasia is characterized by a shallow acetabulum that does not sufficiently cover the head of the femur, leading to instability of the hip and high levels of mechanical stress around the rim of the acetabulum [1]. In a study investigating risk factors for hip joint osteoarthritis (OA), hip dysplasia was found to be a significant risk factor for the development of OA in both men and women, with age being an additional risk factor in women [2]. Hip dysplasia can present either in infancy, termed developmental dysplasia of the hip (DDH), or later in young adulthood, termed acetabular dysplasia. Females have been known to have a much higher risk of hip dysplasia compared to their male counterparts; however, males with hip dysplasia have higher incidences of concomitant hip deformities, which may lead to the development of femoroacetabular impingement [1]. The lateral-center edge angle (LCEA) is a commonly used radiographic measurement to measure the severity of hip dysplasia. The LCEA is formed by two lines from the center of the head of the femur to the lateral edge of the acetabular roof and another line directly vertical from the femoral head. An LCEA of ≥25° is considered normal, <20° is consistent with hip dysplasia, and values in between are considered transitioning to hip dysplasia [3].

Less invasive treatment options for hip dysplasia include hip arthroscopy, but a recent systematic review of 33 studies concluded that isolated arthroscopic treatment should be reserved for patients with borderline hip dysplasia, as poor outcomes were seen in the moderate and severe hip dysplasia groups [4]. Another study, however, reported a high dissatisfaction rate (40%) of 47 borderline dysplastic hips undergoing primary hip arthroscopy [5]. The most appropriately indicated procedure to correct hip dysplasia, however, is the periacetabular osteotomy (PAO), a hip preservation surgery introduced by Ganz et al., in which fluoroscopically guided osteotomies around the pelvis are made to allow for repositioning of the acetabulum to fit properly on the femoral head. Screws are initially placed to support the repositioned acetabulum, and over time, bone growth occurs in the spaces where the osteotomies were performed [6]. In patients with hip dysplasia, when there is a severe progression of untreated osteoarthritis leading to significant loss of function, total hip arthroplasty (THA) is the recommended surgical treatment option, as the PAO is often unsuccessful in the long-term preservation of the hip joint in these patients [7].

This systematic review reports factors such as preoperative Tönnis osteoarthritis grade, pre- and postoperative LCEA, and preoperative hip joint congruency that impact patient-reported outcome measurements to determine the success of the PAO in our included studies. This review also reports commonly used patient-reported outcome measurements such as the Harris Hip Score (HHS) and the Western Ontario and McMaster Universities Arthritis Index (WOMAC) to further analyze the success of the PAO in our included studies.

## Review

Methods

This systematic review followed the Preferred Reporting Items for Systematic Review and Meta-Analyses (PRISMA) guidelines. A literature search for this systematic review was performed on the PubMed, Scopus, and Embase databases. Our search strategy included various combinations of the keywords "periacetabular osteotomy," "outcome," "Harris Hip Score," and "WOMAC" within the title and abstract of articles.

The exclusion criteria were the following: articles in a different language, case reports, articles detailing surgical techniques, review articles, articles in which patients underwent prior surgical intervention for symptomatic hip dysplasia, articles with incomplete data on patient-reported outcomes, case series of fewer than 10 patients, and non-human studies. We included articles in which the PAO was performed in patients with no prior intervention for symptomatic hip dysplasia and in which both preoperative and postoperative patient-reported outcomes were reported. Articles that reported either the HHS, modified Harris Hip Score (mHHS), or WOMAC were included [8-14].

**Figure 1:**
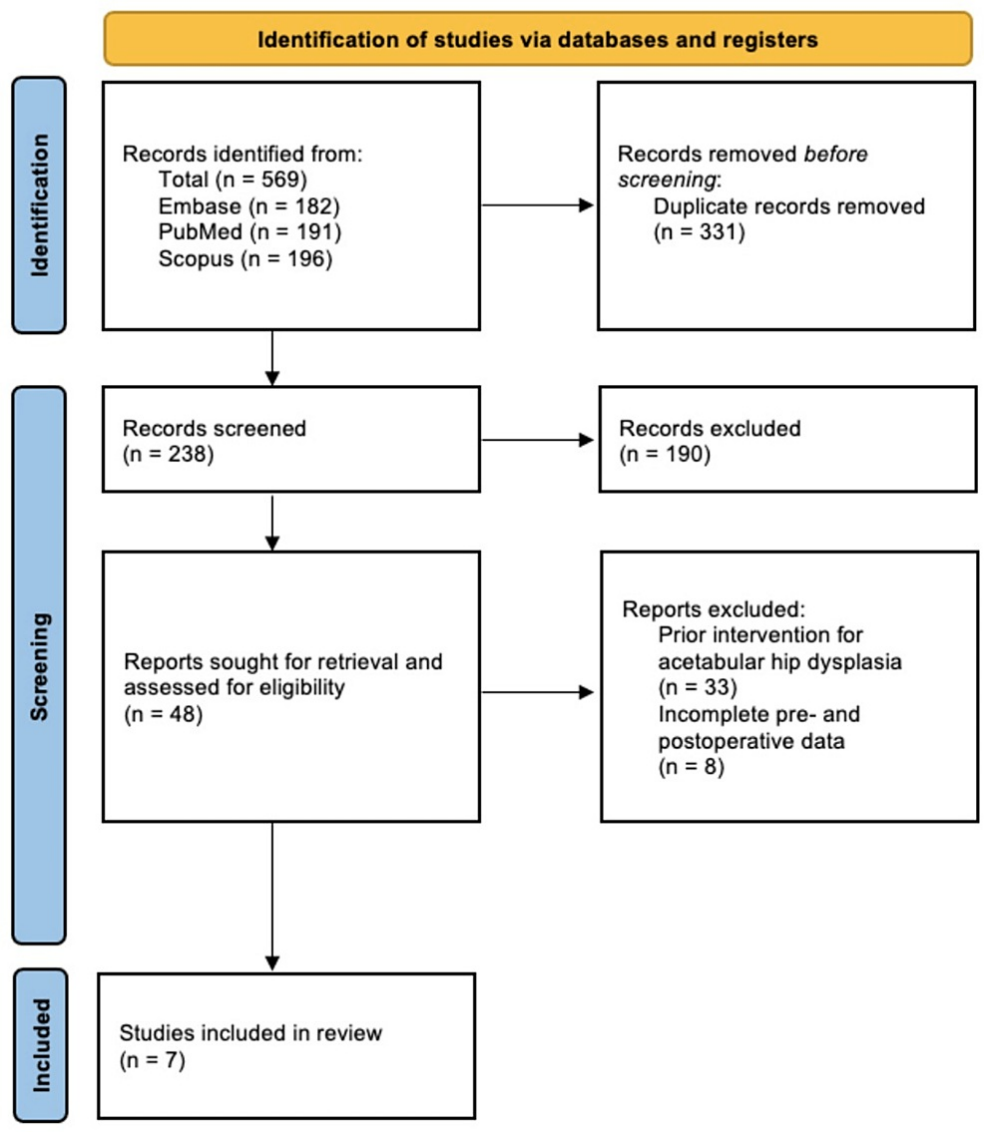
PRISMA diagram illustrating the article selection process PRISMA: Preferred Reporting Items for Systematic Reviews and Meta-Analyses.

This search yielded 569 studies, out of which 331 duplicates were removed. Screening of the title and abstract of the remaining 238 studies resulted in 190 being excluded due to irrelevance to the topic of patient-reported outcomes for the PAO in patients with symptomatic hip dysplasia. A full-text assessment was done on 48 studies, of which 41 were excluded based on our established exclusion criteria. The seven remaining studies were included in this systematic review. Figure 1 illustrates our article selection process, according to the PRISMA guidelines used in our study. Identification of studies was done with the consensus of two authors, and when an agreement could not be reached, a third author was consulted.

The following data were extracted from all seven included articles: first author, article title, time range of the study, study type, number of patients, gender of patients, age of patients, number of hips, mean follow-up time, BMI, LCEA, anterior center-edge angle (ACEA), acetabular index (AI), Tönnis osteoarthritis grade, mHHS, HHS, WOMAC, indications for surgery, complications, outcomes, and patient satisfaction. Data were extracted by two authors independently, and differences were resolved through extensive discussion. Additionally, a quality assessment of the included articles was done by two authors independently using the Methodological Index of Non-Randomized Studies (MINORS) grading system [15], and disagreements were resolved through extensive discussion.

Results

Demographic Data

Across the eight studies, there were a total of 334 patients with a total of 366 hips. The mean age of patients was 35.8 years, with the range of ages being between 12 and 60 years old (Table 1).

**Table 1:** Patient demographic information and article MINORS Quality Assessment Score SD: standard deviation, MINORS: Methodological Index of Non-Randomized Studies grading system, NR: not reported.

Author	Number of patients (female/male)	Number of hips	Mean age in years (range)	Mean follow-up time ± SD (range)	MINORS Score
Fan et al. [8]	59 (42/17)	66 (NR)	33.61 (18-54)	3.01 years ± 1.19 (1-6 years)	15
Fujita et al. [9]	83 (79/4)	85 (81/4)	41.2 (20-60)	12.4 months (12-16 months)	9
Gu et al. [10]	44 (40/4)	44 (40/4)	31.2 ± 9.4 (12-49)	18 months (12-27 months)	12
Maeyama et al. [11]	25 (24/1)	25 (24/1)	32.1 (14-56)	1 year for all	14
Millis et al. [12]	70 (NR)	87 (NR)	43.6 (40-51)	4.9 years (2-13 years)	13
Sakamoto et al. [13]	27 (27/0)	33 (NR)	17 (14-19)	33.3 months (24-96 months)	14
Kralj et al. [14]	26 (22/4)	26 (NR)	34 (18-50)	12 years (7-15 years)	14

Radiographic Data, Patient-Reported Outcomes, and Complications

The most commonly reported preoperative and postoperative radiographic measurements were the LCEA, and of studies that mentioned the LCEA, the mean preoperative LCEA was 9.97° and the mean postoperative LCEA was 35.4°. The preoperative Tönnis osteoarthritis grade of the studies that reported it was as follows: 0 (84 hips), 1 (123 hips), 2 (47 hips), and 3 (1 hip). The postoperative Tönnis osteoarthritis grade of the studies that reported it was as follows: 0 (41 hips), 1 (47 hips), 2 (10 hips), and 3 (1 hip). Of the patient-reported outcomes mentioned, five studies reported HHS, one study reported mHHS, and one study reported WOMAC. Of studies reporting HHS, the mean preoperative HHS was 68.92 and the mean postoperative HHS was 89.1. Of the study that reported mHHS, the mean preoperative mHHS was 70 and the mean postoperative mHHS was 91. Of the studies reporting WOMAC, the mean preoperative WOMAC was 66 and the mean postoperative WOMAC was 63. Lateral femoral cutaneous nerve (LFCN) damage was the most common complication, occurring in 37 hips (Table 2).

**Table 2:** Radiographic data, patient-reported outcomes, and complications Mean ± SD (range) reported for radiographic findings and patient-reported outcomes when available. NR: not reported.

Author	Preoperative radiographic findings	Postoperative radiographic findings	Preoperative Tönnis osteoarthritis grade	Postoperative Tönnis osteoarthritis grade	Preoperative patient-reported outcome	Postoperative patient-reported outcome	Complications (incidence)
Fan et al. [8]	LCEA: 9.02° ± 13.08°, AI: 22.82° ± 9.45°	LCEA: 38.02° ± 8.28°, AI: 0.07° ± 7.13°	0 (1 hip), 1 (40 hips), 2 (24 hips), 3 (1 hip)	NR	HHS: 61.00 ± 16.16	HHS: 80.65 ± 7.14	Infection (2 hips), non-union of fracture (1 hip), minor nerve damage (10 hips)
Fujita et al. [9]	NR	LCEA: 40.66°, ACEA: 42.99°, AI: 0.35°	NR	Improved 0 to 1 (5 hips), 2 to 3 (2 hips)	HHS: 75.5 (39–96)	HHS: 94.2 (70–100)	NR
Gu et al. [10]	LCEA: 10.7° ± 7.2°, AI: 23°	LCEA: 34.8° ± 7.1°, AI: 9.5°	0 (10 hips), 1 (32 hips), 2 (2 hips)	0 (9 hips), 1 (34 hips), 2 (2 hips)	mHHS: 70	mHHS: 91	Sensory disturbance of the lateral femoral cutaneous nerve (6 hips)
Maeyama et al. [11]	LCEA: 10.68° ± 7.42°	LCEA: 29.8° ± 6.32°	0 or 1 for all	NR	HHS: 78.08 (47–96)	HHS: 95.36 (88–100)	NR
Millis et al. [12]	LCEA: 8.1° ± 7.6° (−12° to 30°), ACEA: 5.2° ± 8.9° (−15° to 26°), AI: 22.3° ± 7.4° (7°–54°)	LCEA: 30.1° ± 10.1° (−1° to 53°), ACEA: 30.4° ± 12.4° (12°–79°), AI: 7.5° ± 6.3° (-10° to 20°)	0 (32 hips), 1 (39 hips), 2 (16 hips)	NR	HHS: 60.2 ± 11.4 (40–79)	HHS: 85.4 ± 17.1 (39–100)	Global nonunion associated with GI sepsis 1 month after surgery (1 hip), sciatic sensory neuropraxia (1 hip)
Sakamoto et al. [13]	LCEA: 10.2° (−3° to 19°)	LCEA: 33.1° (17°–55°)	0 (29 hips), 1 (3 hips), 2 (1 hip)	0 (28 hips), 1 (4 hips), 2 (1 hip)	HHS: 80.1 (45–90)	HHS: 95.4 (85–100)	Symptomatic ischial nonunion (1 hip), nonunion of the superior ramus osteotomy (4 hips), superficial stitch abscess (2 hips), transient lateral femoral cutaneous nerve palsy (3 hips)
Kralj et al. [14]	LCEA: 15° (7°–26°), ACEA: 22° (5°–40°)	LCEA: 37° (20°–68°), ACEA: 38° (21°–56°)	0 (12 hips), 1 (9 hips), 2 (4 hips)	0 (4 hips), 1 (9 hips), 2 (7 hips), 3 (2 hips)	WOMAC: 66	WOMAC: 63	Wound hematoma (1 hip), fracture of the acetabulum (1 hip), pulmonary embolism (1 patient), sciatic nerve dysfunction (1), dysaesthesia of lateral femoral cutaneous nerve (18)

Factors Associated with Outcomes

Fan et al. determined that changes in preoperative and latest follow-up HHS less than nine were defined as an adverse outcome. Based on this definition, 46 hips were preserved while 20 were symptomatic. The mean postoperative HHS of the preserved and symptomatic groups was 86.02 ± 3.29 and 74.06 ± 5.99, respectively, a statistically significant difference. Statistical analysis determined that failure was much more likely when the postoperative LCEA was greater than 38° or if the hips had fair or poor preoperative joint congruency. Patients with an oversized postoperative LCEA and poor or fair preoperative hip joint congruency were correlated with poor postoperative patient-reported outcomes via the HHS. A Tönnis angle of −10° to 0° was however determined to be a factor correlated with success, as evidenced by a satisfactory postoperative HHS [8].

Fujita et al. assessed the success of the PAO based on the rate of return to work one year post-operation. The rate of return to work was 82.4%, with eight hips being unable to return to work due to symptoms regarding their hip. No significant differences in clinical parameters or a specific type of work were determined between patients returning to work and those not returning to work one year post-operation. Additionally, there were no conversions to THA [9].

Gu et al. found that the probability of the postoperative mHHS being classified as excellent was six times more likely if a patient’s hip joint had a preoperative LCEA of greater than 4.5° compared to less than 4.5°. This finding of a higher postoperative mHHS in those with a preoperative LCEA of greater than 4.5° was most common in patients with a preoperative Tönnis osteoarthritis grade of 1. Additionally, there were decreased levels of postoperative pain and symptoms and no cases of failed surgeries [10].

Maeyama et al. studied the dynamic instability of hips using triaxial accelerometry after undergoing PAO, a unique measurement of outcome different from other studies in this review. The findings suggest that PAO reduces dynamic instability, provides pain relief, and improves acetabular coverage in patients with hip dysplasia [11].

Millis et al. performed a survival analysis of patients undergoing the PAO and found that, of all 87 patients, the mean survival was 8.4 years. 66 (75.9%) of the 87 patients were found to maintain their native hip joint at a mean follow-up of five years. The remaining 21 hips underwent THA at a mean time of 5.2 years (range, 1.9-7.6 years) from their initial PAO. The risk for an earlier conversion to THA was correlated with a higher preoperative Tönnis osteoarthritis grade. At five years, the risk of conversion to THA was 12% in hips with a preoperative Tönnis osteoarthritis grade of zero or one and 27% for a Tönnis osteoarthritis grade of two. Additionally, preoperative HHS for both surviving and converting to THA hips was similar: 60.7 and 57.6, respectively. Postoperatively, however, surviving hips had a significantly higher HHS compared to hips that were converted to THA: 90.3 and 61.9, respectively [12].

Kralj et al. also performed a survivorship analysis of the PAO to determine the long-term mechanical status of the hip. Of 26 total hips, four required conversion to THA after a mean time of 4.5 years from the initial PAO. Of the four failed hips, the mean preoperative WOMAC score was 50, the mean preoperative Tönnis osteoarthritis grade was either 2 or 3, and there was a less favorable correction for LCEA and ACEA. Additionally, the four failed hips had higher levels of postoperative peak contact stress compared to the eight hips with advanced arthrosis; both of these groups had similar postoperative LCEA values. The 22 surviving hips, however, had a mean preoperative WOMAC score of 70 and Tönnis osteoarthritis grades of zero or one, whereas the mean postoperative WOMAC was 63, a statistically insignificant difference. Preoperative WOMAC scores and Tönnis osteoarthritis grades were therefore considered very important factors when predicting the long-term outcome of the PAO. Additionally, of the 22 surviving hips, eight hips showed signs of significant arthrosis progression, whereas the remaining 14 hips had either no or mild arthrosis at follow-up. With a relatively long follow-up of 7-15 years, the progression of arthrosis by approximately one grade occurred in all patients, even those considered successful. An even longer follow-up was therefore hypothesized to show clinical deterioration in even the 14 hips considered successful. One factor that may have biased the results was that the average age at operation was lower in those with mild or no arthrosis compared to those with advanced arthrosis. However, since the total number of hips analyzed in this study was only 26, larger case series are necessary to determine whether age is also a risk factor for poor outcomes [14].

Discussion

The PAO as described by Ganz et al. has been considered an effective approach for correcting hip dysplasia [6]. This review provides a compilation of patient-reported outcomes, radiographic characteristics, and outcomes of patients undergoing the PAO for symptomatic acetabular hip dysplasia. The patients in this review did not undergo any prior intervention for acetabular hip dysplasia, allowing for an unbiased reporting of outcomes from all included studies. The minimal clinically important difference (MCID) for HHS in hip preservation surgery has previously been established to be between a seven- and nine-point increase [16]. All five of our studies reporting the HHS had a mean improvement greater than the established MCID value, with a minimum mean increase of 15 points and a maximum mean increase of 25 points. The MCID for the WOMAC score in hip preservation surgery has also previously been established to be between a change of 9 and 12 points from baseline [16]. One of our studies reporting WOMAC achieved an MCID with a 29-point improvement; however, the other study did not achieve an MCID, as the WOMAC score improved by only three points. The MCID for the mHHS in hip preservation has been established to be an improvement of eight points [17]. The single study in our review reporting mHHS reported a mean improvement of 21 points. Overall, seven of our eight included studies achieved an MCID in patient-reported outcomes; however, the single study by Kralj et al. [14] that did not achieve an MCID had a mean follow-up time of 12 years (ranging from 7-15 years). The seven other studies all had a mean follow-up time of fewer than five years. This is a significant finding because Kralj et al. found that of the 22 surviving hips, eight showed signs of significant arthrosis progression, and the remaining 14 showed either no or mild arthrosis. With a relatively long follow-up of 7-15 years, progression of arthrosis by approximately one grade occurred in all patients, even those considered successes, and this is likely the reason for an MCID not having been achieved.

Studies included in this review reporting factors associated with failure include fair or poor hip joint congruency, a postoperative Tönnis angle between −10° and 0°, a postoperative LCEA less than 38°, a preoperative LCEA greater than 4.5°, and a preoperative Tönnis osteoarthritis grade of 0 or 1. Failure was defined as conversion to THA or failure to reach a postoperative patient-reported outcome threshold score. These findings are similar to other studies such as Harting-Andreasen et al., who found that at a 4- to 12-year follow-up, factors associated with conversion to THA included higher age, a preoperative Tönnis osteoarthritis grade of 2, hip incongruency, a postoperative joint space width of 3 mm or less, and a postoperative LCEA less than 30° or greater than 40° [18]. Wells et al. also found that factors impacting progression to THA include age greater than 25 years, poor or fair hip congruency, and a preoperative joint space width less than 2 mm and greater than 5 mm [19].

A limitation of this review was that we did not exclude studies based on the ages of patients undergoing PAO. The effect of this is specifically evident in the findings of the studies by Millis et al. [12], which focused on patients between the ages of 40 and 51, and Sakamoto et al. [13], which focused on patients between the ages of 14 and 19. Despite both groups having similar mean preoperative and postoperative LCEAs, Millis et al. had a significantly higher proportion of hips with a Tönnis osteoarthritis grade of one or two (63%) compared to Sakamoto et al. (12%). Both groups also had significant improvements in the HHS; however, Sakamoto et al. had a mean preoperative HHS of 20 points higher than Millis et al. (80.1 vs. 60.2) and a mean postoperative HHS of 10 points higher (95.4 vs. 85.4). Additionally, Millis et al. reported conversions to THA occurring in 24% of hips. Conversely, the mean age of patients in Fujita et al. [9] and Millis et al. was similar (41.2 vs. 43.6), but Fujita et al. had a postoperative HHS similar to that of Sakamoto et al. (94.2 vs. 95.4). Similar postoperative HHS in Fujita et al. and Sakamoto et al. may, however, be attributed to a shorter follow-up time of 12.4 months in the former and a longer follow-up time of 33.3 months in the latter. In a study by Muffly et al., at a mean follow-up of 4.71 years, preoperative WOMAC scores were the lowest in patients over the age of 40 but were significantly higher postoperatively when compared to patients in groups of less than 20, 20-29, and 30-39 years [20]. These findings thus demonstrate that age alone may not be an appropriate selection criterion to evaluate candidates for the PAO and instead a multitude of variables need to be considered, such as radiographic and physical exam findings.

Of the complications occurring in patients undergoing the PAO in our review, the most common were sensory disturbance of the LFCN, occurring in 37 (10%) hips, and nonunion, occurring in seven (1.9%) hips. In a systematic review discussing complications, Ali et al. found that across 4070 hips in 40 studies, the most common complication was also injury of the LFCN, occurring in 250 (6.14%) hips. Nonunion was the third most common complication, occurring in 90 (2.2%) hips. Increased rates of LFCN injuries were found in patients who underwent the ilioinguinal and two-incision approaches, while minimally invasive trans-trochanteric and trans-sartorial approaches were not associated with nerve injuries [21]. Other studies report LFCN injury occurring in 14.8% to as many as 67% of patients [22-23]. Kalhor et al. and Thiagarajah et al. present similar techniques to avoid injury to the LFCN by initially making a c-shaped incision and then making a fascial incision more laterally or over the belly of the tensor fascia lata to avoid LFCN damage [24-25]. Cates et al., however, believe that the approach of making an incision lateral to the tensor fascia lata may result in damage to the superior or posterior branches of the LFCN crossing the tensor fascia lata [23]. Injury to the LFCN is demonstrated to be exclusively related to the surgical approach, but injury has not been shown to impact hip function and rather has a greater impact on mental health. However, unintended damage to the LFCN is rarely the cause of poor patient outcomes [24,26].

## Conclusions

In patients with no prior intervention for hip dysplasia, the PAO is a successful procedure with significant improvement in postoperative patient-reported outcomes, including the HHS, mHHS, and WOMAC. In this review, patients in six of the seven studies achieved an MCID in patient-reported outcomes. Additionally, studies included in this review report factors associated with failure that include fair or poor hip joint congruency, a postoperative Tönnis angle between −10° and 0°, a postoperative LCEA less than 38°, a preoperative LCEA greater than 4.5°, and a preoperative Tönnis osteoarthritis grade of zero or one. At a long enough follow-up of around 15 years, PAOs initially considered successful may begin to show signs of hip arthrosis progression. Studies with longer follow-ups are prompted to derive firm conclusions regarding the long-term survivorship of the PAO in patients with no prior intervention for hip dysplasia.

## References

[1] Pun S (2016). Hip dysplasia in the young adult caused by residual childhood and adolescent-onset dysplasia. Curr Rev Musculoskelet Med.

[2] Jacobsen S, Sonne-Holm S (2005). Hip dysplasia: a significant risk factor for the development of hip osteoarthritis. A cross-sectional survey. Rheumatology (Oxford).

[3] Gala L, Clohisy JC, Beaulé PE (2016). Hip dysplasia in the young adult. J Bone Joint Surg Am.

[4] Adler KL, Giordano BD (2019). The utility of hip Arthroscopy in the setting of acetabular dysplasia: a systematic review. Arthroscopy.

[5] Yoon SJ, Lee SH, Jang SW, Jo S (2019). Hip arthroscopy of a painful hip with borderline dysplasia. Hip Pelvis.

[6] Ganz R, Klaue K, Vinh TS, Mast JW (1988). A new periacetabular osteotomy for the treatment of hip dysplasias. Technique and preliminary results. Clin Orthop Relat Res.

[7] Yang S, Cui Q (2012). Total hip arthroplasty in developmental dysplasia of the hip: Review of anatomy, techniques and outcomes. World J Orthop.

[8] Fan Y, Li W, Wu Y (2021). The association the patient-reported outcomes after periacetabular osteotomy with radiographic features: a short-term retrospective study. J Orthop Surg Res.

[9] Fujita J, Doi N, Kinoshita K, Sakamoto T, Seo H, Yamamoto T (2022). Rate of return to work after periacetabular osteotomy and Its influencing factors. J Bone Joint Surg Am.

[10] Gu YG, Shi ZW, Yue YH (2021). Analysis of factors affecting early functional recovery of Bernese periacetabular osteotomy. Orthop Surg.

[11] Maeyama A, Naito M, Moriyama S, Yoshimura I (2009). Periacetabular osteotomy reduces the dynamic instability of dysplastic hips. J Bone Joint Surg Br.

[12] Millis MB, Kain M, Sierra R (2009). Periacetabular osteotomy for acetabular dysplasia in patients older than 40 years: a preliminary study. Clin Orthop Relat Res.

[13] Sakamoto T, Naito M, Nakamura Y (2015). Outcome of peri-acetabular osteotomy for hip dysplasia in teenagers. Int Orthop.

[14] Kralj M, Mavcic B, Antolic V, Iglic A, Kralj-Iglic V (2005). The Bernese periacetabular osteotomy: clinical, radiographic and mechanical 7-15-year follow-up of 26 hips. Acta Orthop.

[15] Slim K, Nini E, Forestier D, Kwiatkowski F, Panis Y, Chipponi J (2003). Methodological index for non-randomized studies (minors): development and validation of a new instrument. ANZ J Surg.

[16] Smith MV, Klein SE, Clohisy JC, Baca GR, Brophy RH, Wright RW (2012). Lower extremity-specific measures of disability and outcomes in orthopaedic surgery. J Bone Joint Surg Am.

[17] Kemp JL, Collins NJ, Roos EM, Crossley KM (2013). Psychometric properties of patient-reported outcome measures for hip arthroscopic surgery. Am J Sports Med.

[18] Hartig-Andreasen C, Troelsen A, Thillemann TM, Søballe K (2012). What factors predict failure 4 to 12 years after periacetabular osteotomy?. Clin Orthop Relat Res.

[19] Wells J, Millis M, Kim YJ, Bulat E, Miller P, Matheney T (2017). SSurvivorship of the Bernese periacetabular osteotomy: what factors are associated with long-term failure?. Clin Orthop Relat Res.

[20] Muffly BT, Zacharias AJ, Jochimsen KN, Duncan ST, Jacobs CA, Clohisy JC (2021). Age at the time of surgery is not predictive of early patient-reported outcomes after periacetabular osteotomy. J Arthroplasty.

[21] Ali M, Malviya A (2020). Complications and outcome after periacetabular osteotomy - influence of surgical approach. Hip Int.

[22] Swarup I, Ricciardi BF, Sink EL (2015). Avoiding complications in periacetabular osteotomy. JBJS Rev.

[23] Cates RA, Boon AJ, Trousdale RT, Douge A, Sierra RJ (2019). Prospective evaluation of lateral femoral cutaneous nerve injuries during periacetabular osteotomy. J Hip Preserv Surg.

[24] Kalhor M, Collado D, Leunig M, Rego P, Ganz R (2017). Recommendations to reduce risk of nerve injury during Bernese periacetabular osteotomy (PAO). JBJS Essent Surg Tech.

[25] Thiagarajah S, Bingham JS, Grammatopoulos G, Witt J (2020). A minimally invasive periacetabular osteotomy technique: minimizing intraoperative risks. J Hip Preserv Surg.

[26] Doi N, Kinoshita K, Sakamoto T, Minokawa A, Setoguchi D, Yamamoto T (2021). Incidence and clinical outcome of lateral femoral cutaneous nerve injury after periacetabular osteotomy. Bone Joint J.

